# Alternative approach of complicated peptic ulcer perforation in recurrent and delayed—two cases

**DOI:** 10.1093/omcr/omaf134

**Published:** 2025-08-20

**Authors:** Ahmed Taha

**Affiliations:** Department of General Surgery, SBU, Bursa Yüksek İhtisas Training and Research Hospital, Mimarsinan Mah. Emniyet Cad. Yıldırım, 16310 Bursa - Turkey

**Keywords:** case report, stomach, duodenoplasty, peptic ulcer, perforation, acute abdomen

## Abstract

Gastric and duodenal ulcer perforation represents one of the most critical causes of acute abdomen, necessitating urgent surgical intervention. While primary repair with omental patch remains the standard treatment for uncomplicated cases, alternative surgical approaches may be preferable in complex or delayed presentations. We illustrate this variability through two distinct cases. *Case I:* A recurrent gastric perforation managed via wedge resection using linear staplers, reinforced with Lembert sutures to ensure staple-line integrity. *Case II:* A delayed duodenal perforation treated with Heineke-Mikulicz duodenoplasty combined with omentoplasty to address tissue edema and mitigate leakage risk. These cases highlight the necessity of adapting surgical strategies to patient-specific factors, such as perforation chronicity, tissue viability, and prior intervention history. Unlike routine repairs, complex scenarios often demand advanced techniques to optimize outcomes and reduce morbidity.

## Introduction

Gastric and duodenal ulcer perforation remains a predominant cause of acute abdomen, necessitating emergent surgical management [1]. Clinical severity intensifies in cases involving delayed diagnosis, delayed intervention, significant comorbidities, recurrent perforations, or concern for malignancy [2, 3]. While primary repair —with or without omental patch reinforcement— remains the standard for hemodynamically stable, uncomplicated cases anatomically complex or high-risk presentations necessitate tailored surgical approaches [3]. In the two cases presented here, conventional interventions proved insufficient for addressing unique clinical challenges, requiring deviation from standardized protocols.

### Case I

A 52-year-old nonsmoker, nonalcoholic, hypertensive female with a history of left hemiplegia secondary to cerebrovascular accident presented to the emergency department with a two-day history of severe abdominal pain, nausea, and generalized weakness. The patient was ambulatory on arrival, was conscious, cooperative, and oriented. Her general condition was moderately compromised, with clinical signs of acute abdomen. Medical records indicated three prior hospitalizations for gastric perforation within the past five years:two managed with omental patch repair and once with a conservative management ‘nasogastric decompression, nil per os (NPO) status, and proton pump inhibitor (PPI) infusion’.

Physical examination revealed a marked abdominal distension with localized tenderness and rebound tenderness in the epigastrium and left upper quadrant. Vital signs: BP(Blood pressure) 115/70 mmHg, temperature 36.9°C.

Routine labs showed normal white blood cell count (WBC) 7.99 × 10^3^/μl, hemoglobin (HGB) 14.1 g/dl, platelets (PLT) 256 × 10^3^/μl, C-reactive protein (CRP) 0.4 mg/l, and neutrophils 79.7%.

An upright abdominal X-ray demonstrated free air under both domes of diaphragm (predominantly right-sided). Abdominal CT confirmed free air adjacent to the liver, stomach, Morrison’s pouch, falciform ligament, and above the spleen; along with a 3 cm pelvic fluid collection. These findings confirmed *recurrent gastric perforation*.

**Figure 1:**
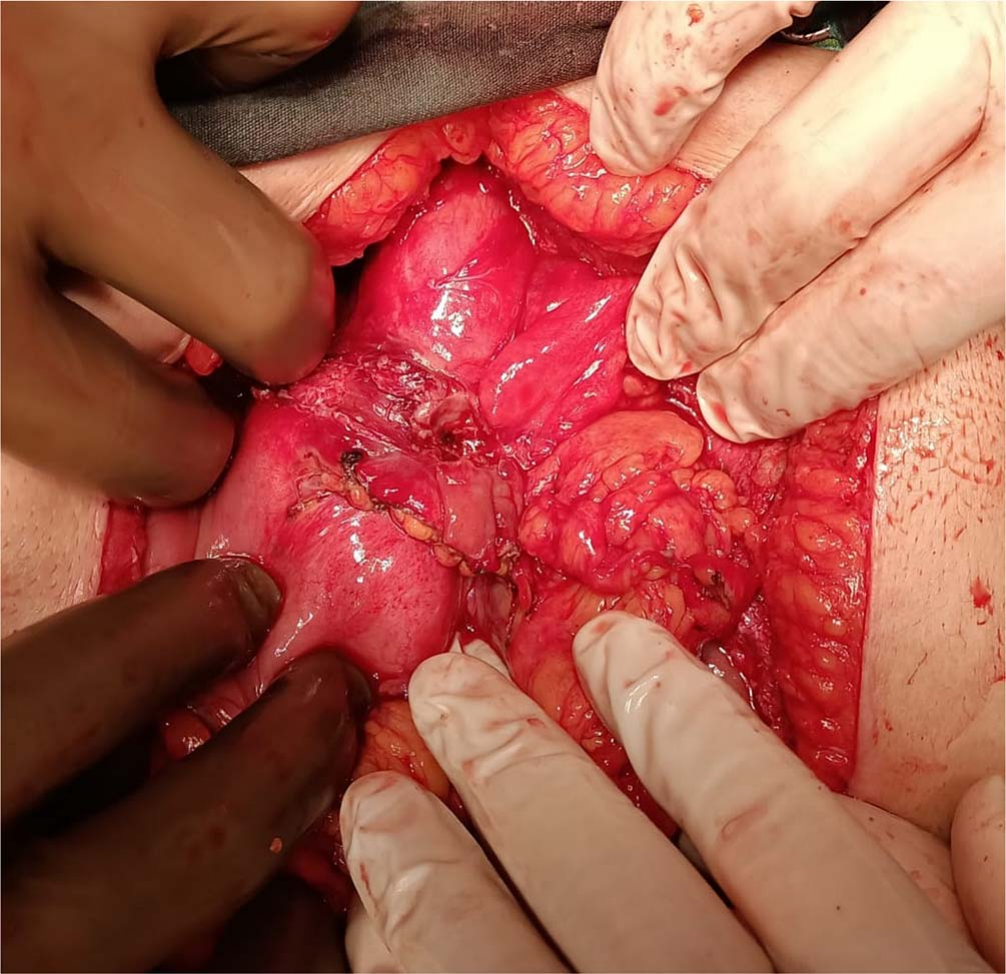
Perforated ulcer defect at the greater curvature.

**Figure 2:**
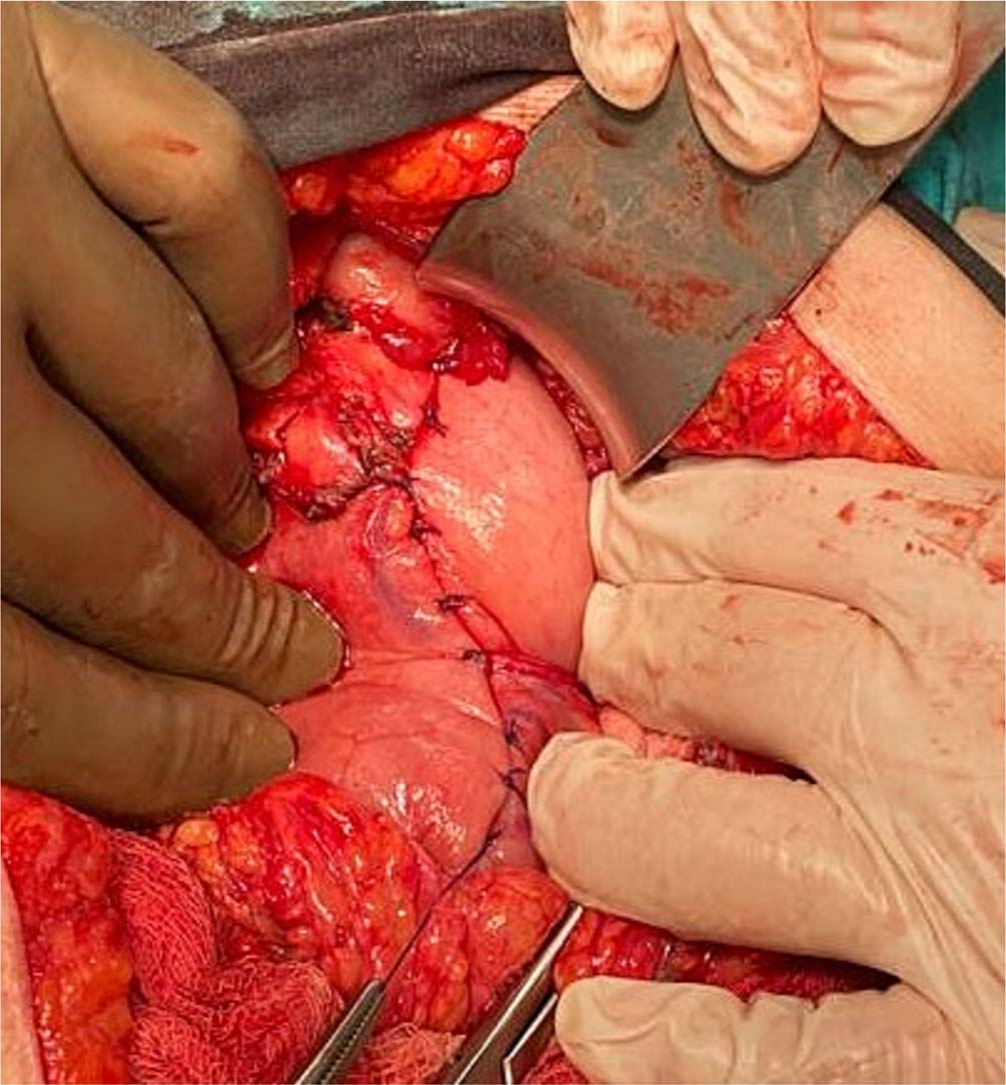
Wedge resection performed using linear staplers, followed by reinforcement with Lembert suture.

Emergency laparotomy via midline upper abdominal incision revealed dense adhesions from prior surgeries. In surgical exploration, we could not proceed from the midline due to dense cysts. It was initiated from the right abdomen under the guidance of the hepatic dome and revealed a five-cm ulcer defect and a two-cm perforation on the greater curvature, six cm proximal to the pylorus (Fig. 1). Disrupted Graham patch repairs from previous interventions were identified. A wedge resection was performed using linear staplers, reinforced with Lembert sutures (Fig. 2). Intraoperative saline lavage was completed, and a drain was placed in the subhepatic space.

Postoperatively, Methylene blue testing on postoperative day 3 confirmed no leakage. The patient was discharged after an uncomplicated 7-day hospitalization. Histopathology revealed a foreign body reaction at the ulcer base, suggesting involvement of prior surgical material.

### Case II

A 69-year-old nonsmoker, nonalcoholic male with poorly controlled type 2 diabetes mellitus (DM), cachexia, and extensive lower extremity dermal wounds and ulcers presented to the emergency department with a five-day history of severe abdominal pain, nausea, vomiting, and generalized weakness. The patient, who lived independently, was ambulatory upon arrival, conscious, cooperative, and oriented. His general condition was moderately compromised, with unequivocal signs due to an acute abdomen. He had no prior surgical history.

Physical examination revealed diffuse abdominal distension, generalized tenderness in all abdominal quadrants with obvious rebound tenderness. Vital signs: BP (Blood pressure) 118/75 mmHg, temperature 36.4°C.

Routine labs showed leukocytosis (WBC 11.93 × 10^3^/μl; neutrophils 71.4%), elevated CRP 15.8 mg/l, renal impairment (BUN 55.4 mg/dl; creatinine 1 mg/dl), and hyperglycemia (glucose 156.4 mg/dl).

An upright abdominal X-ray showed free air under the right dome of the diaphragm. Ultrasound (USG) reported concentric gastric wall thickening at the antrum and the pyloroduodenal junction, with a 20 × 15 mm cystic lesion (suspected closed perforation vs. duodenal diverticulum). Minimal free fluid in perihepatic, perisplenic, and pelvic regions; periportal air bubbles seen in CT scan. Accordingly, the diagnosis of *gastric/duodenal perforation* was made, but the patient initially refused surgery treatment and left the hospital against medical advice.

Four days later, the patient re-presented to the emergency room with critical vital signs, hypotension (85/60 mmHg), hypothermia (35.4°C), and septic shock. Repeat labs showed leukopenia (WBC 3.86 × 10^3^/μl; neutrophils 83.4%), markedly elevated CRP 266.6 mg/l, pre-renal acute kidney injury (BUN 180 mg/dl; creatinine 2.12 mg/dl), electrolyte derangements (Hyponatremia Na 133 mEq/l, hypochloremia Cl 93.4 mEq/l), and coagulopathy (PT 17.6 s; INR 1.51).

Abdominal CT showed diffuse subdiaphragmatic free air, a ten cm pelvic fluid collection, mesenteric edema, and small bowel distension (subileus). The diagnosis of *delayed gastrointestinal perforation complicated by septic shock* was made, and the patient and his family agreed to surgical intervention.

Emergency laparotomy via midline incision revealed feculent intra-abdominal fluid and a four cm post-pyloric perforation with necrotic margins. Necrotic tissue debridement was followed by Heineke-Mikulicz duodenoplasty and omentoplasty. Two drains were placed, one in the subhepatic and the other in the rectovesical space.

The patient was postoperatively intubated and extubated on the second postoperative day. Enteral feeding was initiated on 7^th^ postoperative day after methylene blue testing confirmed repair integrity. He was discharged on 12^th^ postoperative day following an uncomplicated recovery.

## Discussion

Gastric and duodenal ulcer perforations typically manifest as small defects (≤5 mm) and are associated with favorable outcomes when managed promptly through early diagnosis, timely surgical intervention, and structured postoperative care [1, 4]. However, delayed presentations, recurrent perforations, and anatomically complex cases necessitate individualized approaches, as infection progression risks life-threatening complications such as diffuse peritonitis and septic shock [5]. While standardized protocols (primary repair with omental patch) remain first-line for uncomplicated cases, adaptive decision-making guided by intraoperative findings and patient-specific factors is critical in high-risk scenarios [6, 7].

Case I: Recurrent perforation at the same site, compounded by dense adhesions from prior surgeries, rendered standard omentoplasty insufficient. Prolonged dense adhesion and fibrotic tissue at the perforation margin precluded durable primary closure. Wedge resection with linear staplers and Lembert suture reinforcement was prioritized to excise nonviable tissue, restore anastomotic integrity, and facilitate histopathological evaluation of the ulcer base. This approach aligns with evidence advocating resection over repair in recurrent perforations to mitigate reoperation risks [2, 8].

Case II: The delayed presentation, extensive necrosis at the perforation site, and the patient’s comorbidities (uncontrolled diabetes, cachexia) necessitated a less radical repair strategy. Heineke-Mikulicz duodenoplasty with omentoplasty was preferred instead of gastrojejunal anastomosis to avoid anastomotic leakage, reduce stenosis risk, and circumvent the morbidity of gastrojejunostomy in a nutritionally compromised patient. This decision reflects surgical principles for the management of necrotic or unstable perforations in individuals with poor general conditions [7].

## Conclusion

The management of complicated and delayed peptic ulcer perforations necessitates individualized surgical approaches, prioritizing anatomic feasibility, tissue viability, and comorbidities over standardized primary repair techniques. As demonstrated, recurrent perforations with fibrotic margins or delayed presentations complicated by necrosis and systemic sepsis mandate advanced strategies—such as wedge resection, duodenoplasty, or reinforced omentoplasty. Adherence to dynamic decision-making algorithms, incorporating intraoperative findings, histopathological analysis, and physiological status, prevents recurrence, reduces morbidity, and optimizes outcomes in high-risk patients. These principles reinforce the necessity for surgeons to integrate evidence-based guidelines with nuanced clinical judgment in complex scenarios.

## Data Availability

All data supporting the findings of this study are available within the paper.
